# Association between long-term air pollution exposure and insulin resistance independent of abdominal adiposity in Korean adults

**DOI:** 10.1038/s41598-022-23324-4

**Published:** 2022-11-09

**Authors:** Seo Eun Hwang, Hyuktae Kwon, Jae Moon Yun, Kyungha Min, Hyun-Jin Kim, Jin-Ho Park

**Affiliations:** 1 Department of Family Medicine, Seoul National University Hospital, Seoul National University College of Medicine, 103 Daehakro, Yeongun-Dong, Jongno-Gu, Seoul, 03080 South Korea; 2 Department of Family Medicine, Seoul National University College of Medicine, Seoul, South Korea; 3 Big Data Center, National Cancer Control Institute, National Cancer Center, 323 Ilsan-Ro, Ilsandong-Gu, Goyang-Si, Gyeonggi-Do 10408 South Korea

**Keywords:** Environmental sciences, Endocrinology, Risk factors

## Abstract

Significant associations between air pollution (AP) and insulin resistance (IR) have been reported in limited populations or certain patient groups, but few studies have addressed this association in the general population, especially in Asians. Although abdominal fat is a major contributor to IR, previous studies have not fully controlled for its effect in the association between AP and IR. We investigated the association between exposure to AP and IR in Korean adults in the general population and whether this association is maintained even after controlling for the effects of abdominal fat, particularly visceral fat. This was a cross-sectional study. Data were obtained for Korean adults who participated in screening health checkups at Seoul National University Health Examination Center from 2006 to 2014. A total of 4251 men and women aged 22–84 years were included. IR was represented by the homeostasis model assessment of insulin resistance (HOMA-IR). Adiposity traits such as visceral adipose tissue (VAT) and subcutaneous adipose tissue areas were measured by computed tomography. We assessed the annual mean concentrations of air pollutants, including particulate matter with an aerodynamic diameter of 10 µm or less (PM_10_), nitrogen dioxide, sulfur dioxide, and carbon monoxide. HOMA-IR was significantly associated with increased annual mean exposure to PM_10_ in both men (*β* = 0.15; 95% CI 0.09, 0.22) and women (*β* = 0.16; 95% CI 0.09, 0.23), and these associations were maintained even after controlling for VAT area (both *p* < 0.05). The adjusted mean HOMA-IR increased gradually with the level of long-term PM_10_ exposure (low, intermediate, and high exposure) (all *p* for trend < 0.001) in the subgroup analysis. After adjusting for possible confounding factors, including VAT area, the annual mean exposure to PM_10_ was significantly associated with the presence of IR in both men (OR 1.18; 95% CI 1.03, 1.35) and women (OR 1.44; 95% CI 1.18, 1.76). Other air pollutants, such as NO_2_, SO_2_ and CO, did not show any significant associations with HOMA-IR or the presence of IR. Persistent exposure to PM_10_ is the main independent risk factor for IR and exhibits a dose-dependent association regardless of visceral fatness in both men and women.

Long-term exposure to air pollution (AP) and insulin resistance (IR) are both well-known risk factors for metabolic diseases, including atherosclerotic cardiovascular disease (ASCVD)^1–6^. The mechanism underlying IR as a risk factor for ASCVD relates to the reduced ability of insulin to take up and utilize glucose, which leads to diabetes and is a major intermediate in ASCVD progression^7^. Abdominal fat, especially when stored as visceral adipose tissue (VAT), also contributes to the development of IR^8–10^. It has been suggested that proinflammatory cytokines released by VAT and a high rate of lipolysis within VAT result in an excess of circulating free fatty acids, which may lead to a chronic, low-grade inflammatory state and reduced insulin sensitivity^11^. On the other hand, other studies have reported that AP exposure is a risk factor for ASCVD, although the mechanism is not yet clear^4–6^. Given the links between AP, IR, and ASCVD, it is important to examine whether the relationship between AP exposure and ASCVD is related to the risk of IR and, if so, whether the association between AP and IR acts independently of the amount of abdominal fat, which is a well-known mediating factor of the development of IR^8–11^.

Indeed, recent epidemiological evidence has shown that exposure to ambient AP is associated with the risk of developing IR^12–18^. A 2018 systemic review and meta-analysis reported a positive correlation between exposure to AP in the form of particulate matter with an aerodynamic diameter ≤ 10 μm (PM_10_) or nitrogen dioxide (NO_2_) and IR, as indicated by the homeostasis model assessment of insulin resistance (HOMA-IR)^19^. Another study conducted in the United States in 2016 reported a positive association between short-term exposure (up to 58 days cumulative lagged averages) to particulate matter with an aerodynamic diameter ≤ 2.5 μm (PM_2.5_) and IR^13^. Other studies in children have also found a similar association between air pollutants and IR^17,20^. These findings may be explained by the fact that inhaled air pollution may induce pulmonary oxidative stress and affect tissue and organ inflammation, which may alter glucose metabolism^5,21,22^. However, most previous studies on AP and IR have been conducted in North America or Europe^12–14,16,17^ The composition of air pollutants differs between Asia and other regions^13,14,16,18,23,24^, and insulin sensitivity also differs between ethnic groups^25–28^. In addition, in some studies, the data have been obtained from specific populations, such as children or patients with diabetes mellitus (DM)^17,24^, and few studies have been conducted on the general population. Importantly, few studies have considered abdominal fatness when examining the association between AP exposure and IR. That is, it is unknown whether long-term AP exposure, which is especially important because the cumulative effect is much larger than that of short-term exposure^29^, leads to IR through an intermediate step of increasing abdominal fatness or whether this process is independent of abdominal fatness. Considering the potential confounding effect of abdominal fatness on IR, it is important to understand whether fat level affects the relationship between long-term AP exposure and IR in the general population.

In this study, we investigated the effects of long-term AP exposure on IR, as represented by HOMA-IR. We included several adiposity traits, including abdominal fat variables, as covariates in a study of a large population of Korean adults.

## Methods

### Study design and participants

This was a cross-sectional study. The study population was recruited from 2006 to 2014 at the two health checkup centers managed by Seoul National University Hospital (SNUH). The population received periodic comprehensive health screenings and completed questionnaires about their medical history, current medication, and lifestyle. A total of 5058 participants who underwent screening health checkups, including abdominal fat computed tomography (CT), were included. We finally included 4251 participants after excluding those who had missing information on address (N = 77) or clinical variables (N = 807), such as serum glucose and insulin levels. The study was approved by the SNUH Institutional Review Board (IRB# 2107-153-1237), and all methods were performed in accordance with relevant guidelines and regulations.

### Assessment of exposure to AP

The concentrations of particulate matter or chemicals in AP were obtained from real-time monitoring data of ambient AP collected from approximately 300 atmospheric monitoring sites nationwide by the Ministry of the Environment of Korea (see Supplementary Figure S1) (https://www.airkorea.or.kr). The data included the concentrations of particulate matter; PM_10_, NO_2_, sulfur dioxide (SO_2_), and carbon monoxide (CO) were measured every 24 h from January 1, 2006, to December 31, 2014. Using residential postcode information for each participant, we identified the monitoring station closest to each participant’s home. To assess the level of long-term exposure to ambient AP for each individual, we calculated the annual average concentrations corresponding to the health checkup year at the monitoring station closest to each participant’s home. To perform the subgroup analysis according to the quantitative PM_10_ exposure level, the PM_10_ concentration was classified into three levels using quartiles: low exposure (quartile 1), intermediate exposure (quartiles 2 and 3), and high exposure (quartile 4).

### Assessment of IR

IR was classified using the HOMA-IR index, which has been shown to be a robust tool for the surrogate assessment of IR^30^. HOMA-IR can be calculated by multiplying fasting serum glucose (mg/dL) and insulin (μU/mL) concentrations and dividing by 405 as follows:^31^

$$HOMA\_IR=\frac{Fasting glucose(\mu U/mL)\times Fasting insulin(mg/dL)}{405}$$

A cutoff value of 2.5 was used to diagnose the presence of IR, as reported previously.^31^ Serum glucose and insulin were measured from blood samples collected after at least 12 h of overnight fasting.

### Assessment of obesity

Obesity was identified according to body mass index (BMI) and VAT and subcutaneous adipose tissue (SAT) areas^8,32^. BMI was calculated as weight (kg) divided by height (m^2^). Total adipose tissue (TAT) and VAT areas were measured at the umbilical level using a Somatom Sensation 16 CT scanner (Siemens AG, Erlangen, Germany). The cross-sectional surface area of abdominal fat was calculated automatically using Rapidia 2.8 CT software (INFINITT, Seoul, Korea)^33^. The VAT area was defined by delineating the intra-abdominal fat bound by the parietal peritoneum or transversalis fascia and excluding the vertebral column and paraspinal muscles^34^. The SAT area was calculated by subtracting the VAT area from the TAT area^33^.

### Potential covariates

To control for the effects of potential confounding variables, we included demographic data on age, sex, smoking status, alcohol consumption, physical activity, and underlying diseases, which are known risk factors for IR development^13–19^. The data were obtained from a questionnaire, physical examination by the family physician, and laboratory test results. Smoking was classified as never, former, or current smoking. Alcohol consumption was categorized as never, former, or current consumption of alcohol. Regular physical activity was defined as engaging in moderate- or vigorous-intensity exercise at least once a week for ≥ 30 min. Blood pressure (BP) was measured with the participant in a sitting position after at least 5 min of rest. Venous blood samples were obtained after a minimum of 14 h of overnight fasting. Hypertension was defined as systolic BP ≥ 140 mmHg or diastolic BP ≥ 90 mmHg or the use of antihypertensive medication. Dyslipidemia was defined as total cholesterol ≥ 240 mg/dL or the use of a lipid-lowering agent.

### Statistical analysis

Baseline characteristics are presented as numbers (%) for categorical variables and means (standard deviations, SDs) for continuous variables. Significant differences were identified using the chi-squared test for categorical variables and a two-tailed Student’s *t* test for continuous variables. To examine associations between HOMA-IR or the presence of IR and obesity traits or AP exposure, multiple linear regression and multiple logistic regression analyses were conducted. The results are presented as the beta (β) coefficient and 95% confidence interval (CI) for HOMA-IR and as the odds ratio (OR) and 95% CI for the presence of IR.

The associations between obesity traits and HOMA-IR or the presence of IR were analyzed in a crude model and an adjusted model, the latter of which included age, smoking status (never, former, or current), alcohol consumption (never, former, or current), regular physical activity (yes or no), hypertension, and dyslipidemia as covariates. The associations between AP exposure and HOMA-IR or the presence of IR were analyzed in a crude model and three adjusted models. Model 1 was adjusted for age, BMI, smoking status, alcohol consumption, regular physical activity, hypertension, and dyslipidemia. Model 2 was adjusted as for Model 1 plus VAT and SAT areas. Model 3 was adjusted as for Model 1 plus VAT area.

To evaluate whether the association between PM_10_ exposure and IR was dose dependent, the adjusted mean HOMA-IR value was calculated according to the stratified levels of PM_10_ exposure using regression analysis in the same models. Pairwise comparisons of the mean values were used to identify groups that differed from each other, and the dose–response relationship between PM_10_ exposure and IR was validated by calculating the *p* for trend. The estimates of air pollutants were obtained by scaling to the interquartile range (IQR), and VAT and SAT areas were modified to express the value per 100 cm^2^ for analyses. All statistical analyses were performed using STATA 16.1.

### Ethics approval and consent to participate

The SNUH Institutional Review Board (IRB number: 2107-153-1237) approved this study. Informed consent was obtained from all subjects and/or their legal guardians.

## Results

### Baseline characteristics

Table 1 shows the detailed characteristics of the participants (N = 4251) according to sex and IR. In the final study population, 3003 (70.64%) were men and 1248 (29.36%) were women, and the prevalence of IR was 21.05% and 18.19%, respectively. In both men and women, the BMI and VAT and SAT areas were significantly higher in participants with IR than in those without (all *p* < 0.001). The mean exposure to PM_10_ in both men and women and exposure to CO in women were significantly higher in participants with IR than in those without (all *p* < 0.01). The median AP exposure levels and IQRs in the total population were 49.33 μm/m^3^ and 11 μm/m^3^ for PM_10_, 30.24 ppb (parts per billion) and 14 ppb for NO_2_, 5.15 ppb and 2 ppb for SO_2_, and 0.57 ppm and 0.2 ppm for CO, respectively (data not shown).

**Table 1:** Characteristics of the study participants.

Characteristics	Men				Women			
	Insulin resistance				Insulin resistance			
	No	Yes	Total	*p* value	No	Yes	Total	*p* value
n	2371 (78.95)	632 (21.05)	3003 (100)		1021 (81.81)	227 (18.19)	1248 (100)	
Age (years), mean ± SD	52.66 ± 8.85	52.59 ± 9.36	52.65 ± 8.96	0.850	55.92 ± 8.61	58.26 ± 8.93	56.35 ± 8.71	< 0.001
BMI (kg/m^2^), mean ± SD	24.02 ± 2.59	26.42 ± 2.88	24.53 ± 2.83	< 0.001	23.02 ± 2.88	25.45 ± 3.28	23.46 ± 3.10	< 0.001
VAT (cm^2^), mean ± SD	123.50 ± 53.48	170.03 ± 59.21	133.30 ± 57.92	< 0.001	84.63 ± 39.86	121.58 ± 49.88	91.35 ± 44.20	< 0.001
SAT (cm^2^), mean ± SD	128.75 ± 49.46	168.6 ± 56.78	137.14 ± 53.6	< 0.001	173.78 ± 62.91	208.86 ± 66.94	180.16 ± 65.06	< 0.001
**PA, n (%)**				< 0.001				< 0.001
No regular PA	1176 (49.60)	375 (59.34)	1551 (51.65)		675 (66.11)	184 (81.06)	859 (68.83)	
Regular PA	1195 (50.40)	257 (40.66)	1452 (48.35)		346 (33.89)	43 (18.94)	389 (31.17)	
**Smoking, n (%)**				0.505				0.271
Never	528 (22.29)	129 (20.41)	657 (21.89)		943 (92.45)	210 (92.92)	1153 (92.54)	
Former	1055 (44.53)	281 (44.46)	1336 (44.52)		31 (3.04)	10 (4.42)	41 (3.29)	
Current	786 (33.18)	222 (35.13)	1008 (33.59)		46 (4.51)	6 (2.65)	52 (4.17)	
**Alcohol consumption, n (%)**				0.610				< 0.001
Never	417 (17.60)	115 (18.20)	532 (17.73)		648 (63.47)	180 (79.30)	828 (66.35)	
Former	167 (7.05)	51 (8.07)	218 (7.26)		53 (5.19)	13 (5.73)	66 (5.29)	
Current	1785 (75.35)	466 (73.73)	2251 (75.01)		320 (31.34)	34 (14.98)	354 (28.37)	
**Hypertension, n (%)**				< 0.001				< 0.001
No	1566 (66.13)	297 (47.07)	1863 (62.12)		714 (70)	109 (48.02)	823 (66)	
Yes	802 (33.87)	334 (52.93)	1136 (37.88)		306 (30)	118 (51.98)	424 (34)	
**Dyslipidemia, n (%)**				0.003				0.062
No	1890 (79.75)	469 (74.21)	2359 (78.58)		775 (75.98)	159 (70.04)	934 (74.9)	
Yes	480 (20.25)	163 (25.79)	643 (21.42)		245 (24.02)	68 (29.96)	313 (25.1)	
**Air pollutants**								
PM_10_ (μm/m^3^), mean ± SD	49.46 ± 7.78	50.45 ± 8.06	49.67 ± 7.85	0.005	49.58 ± 8.36	52.66 ± 9.49	50.14 ± 8.65	< 0.000
Median (IQR)	49.05 (10)	50.25 (10)	49.37 (10)		48.7 (11)	52.61 (14)	48.97 (12)	
NO_2_ (ppb), mean ± SD	30.62 ± 12.38	30.75 ± 12.86	30.65 ± 12.48	0.815	29.35 ± 12.07	29.76 ± 12.16	29.42 ± 12.08	0.645
Median (IQR)	31.38 (14)	31.39 (15)	31.38 (14)		29.94 (15)	30.43 (16)	29.95 (15)	
SO_2_ (ppb), mean ± SD	5.1 ± 1.42	5.03 ± 1.49	5.08 ± 1.43	0.272	0.05 ± 5.19	5.47 ± 1.65	5.31 ± 1.55	0.086
Median (IQR)	4.94 (2)	4.84 (2)	4.92 (2)		5.2 (2)	5.27 (2)	5.22 (2)	
CO (ppm), mean ± SD	0.57 ± 0.14	0.58 ± 0.15	0.57 ± 0.14	0.121	0.57 ± 0.14	0.6 ± 0.17	0.57 ± 0.15	0.009
Median (IQR)	0.56 (0.2)	0.57 (0.2)	0.56 (0.2)		0.56 (0.2)	0.58 (0.2)	0.56 (0.2)	

*SD* standard deviation, *BMI* body mass index, *VAT* visceral adipose tissue, *SAT* subcutaneous adipose tissue, *PA* physical activity, *PM*_*10*_ particulate matter with aerodynamic diameter ≤ 10 μm, *IQR* interquartile range, *NO*_*2*_ nitrogen dioxide, *SO*_*2*_ sulfur dioxide, *CO* carbon monoxide.

Data are presented as the mean ± SD for continuous variables or n (%) for categorical variables; *p* values were calculated using the chi-squared test for categorical variables and Student’s *t* test for continuous variables.

### Associations between obesity traits and HOMA-IR or the presence of IR

The associations between obesity traits such as BMI and VAT and SAT areas and IR, represented as HOMA-IR level and presence of IR (HOMA-IR ≥ 2.5), are presented in Table 2. All obesity traits in men and women were positively associated with both HOMA-IR and the presence of IR even after adjustment for all possible confounding variables. Among the obesity traits, an increase of 100 cm^2^ in VAT area showed the strongest association with both increased HOMA-IR (*β* = 0.89; 95% CI = 0.80, 0.97) and elevated odds for the presence of IR (OR = 4.00; 95% CI = 3.34, 4.78) in men. The positive correlations were even stronger between VAT area and HOMA-IR (*β* = 0.96; 95% CI = 0.83, 1.10) or the presence of IR (OR = 5.93; 95% CI = 4.03, 8.72) than between BMI or SAT area and HOMA-IR or the presence of IR in women.

**Table 2:** Results of the regression analysis of the associations between HOMA-IR or the presence of insulin resistance and obesity traits.

Characteristics	HOMA-IR				Presence of insulin resistance			
	Crude		Adjusted Model^a^		Crude		Adjusted Model^a^	
	*β* (95% CI)	*p* value	*β* (95% CI)	*p* value	OR (95% CI)	*p* value	OR (95% CI)	*p* value
**Total**								
BMI (kg/m^2^)	0.17 (0.16, 0.19)	< 0.001	0.16 (0.15, 0.18)	< 0.001	1.35 (1.31, 1.39)	< 0.001	1.33 (1.29, 1.37)	< 0.001
VAT (cm^2^)	0.87 (0.81, 0.94)	< 0.001	0.87 (0.80, 0.93)	< 0.001	3.95 (3.43, 4.54)	< 0.001	4.06 (3.48, 4.74)	< 0.001
SAT (cm^2^)	0.62 (0.56, 0.68)	< 0.001	0.61 (0.55, 0.68)	< 0.001	2.63 (2.32, 2.98)	< 0.001	2.73 (2.38, 3.13)	< 0.001
**Men**								
BMI (kg/m^2^)	0.20 (0.18, 0.22)	< 0.001	0.19 (0.17, 0.21)	< 0.001	1.39 (1.34, 1.44)	< 0.001	1.37 (1.32, 1.42)	< 0.001
VAT (cm^2^)	0.93 (0.85, 1.01)	< 0.001	0.89 (0.80, 0.97)	< 0.001	4.19 (3.54, 4.97)	< 0.001	4.00 (3.34, 4.78)	< 0.001
SAT (cm^2^)	0.93 (0.84, 1.01)	< 0.001	0.88 (0.79, 0.97)	< 0.001	4.03 (3.37, 4.83)	< 0.001	3.89 (3.23, 4.70)	< 0.001
**Women**								
BMI (kg/m^2^)	0.13 (0.11, 0.14)	< 0.001	0.11 (0.09, 0.13)	< 0.001	1.29 (1.23, 1.35)	< 0.001	1.28 (1.21, 1.35)	< 0.001
VAT (cm^2^)	1.02 (0.89, 1.15)	< 0.001	0.96 (0.83, 1.10)	< 0.001	6.16 (4.37, 8.68)	< 0.001	5.93 (4.03, 8.72)	< 0.001
SAT (cm^2^)	0.39 (0.30, 0.48)	< 0.001	0.33 (0.24, 0.42)	< 0.001	2.22 (1.78, 2.77)	< 0.001	2.14 (1.69, 2.70)	< 0.001

*HOMA-IR* homeostasis model assessment of insulin resistance index, *BMI* body mass index, *VAT* visceral adipose tissue, *SAT* subcutaneous adipose tissue, *OR* odds ratio, *CI* confidence interval.

The beta coefficients, ORs, and 95% CIs for VAT and SAT areas were scaled to 100 cm^2^.

^a^Adjusted model included age, smoking status (never, former, or current smoking), alcohol consumption (never, former, or current consumption), regular physical activity (yes or no), hypertension, and dyslipidemia as covariates.

### Associations between long-term exposure to AP and HOMA-IR

Table 3 shows the results of the linear regression analysis of the associations between long-term exposure to AP and HOMA-IR, which reflects the extent of IR. A higher IQR for PM_10_ was associated with an increase in HOMA-IR in both men and women. The significance was sustained in the adjusted models. In Model 3, including VAT as a covariate, the HOMA-IR score increased by 14% (95% CI = 0.08, 0.21) and 14% (95% CI = 0.07, 0.21) as the IQR (11 μg/m^3^) for PM_10_ exposure increased in men and women, respectively. These values were similar to those in Model 2, which adjusted both VAT and SAT: 15% (95% CI = 0.08, 0.21) and 14% (95% CI = 0.07, 0.21) in men and women, respectively. CO correlated positively with the HOMA-IR score in the crude model, but this association was no longer significant in men or women after adjusting for other variables. Exposure to the other pollutants was not significantly associated with HOMA-IR.

**Table 3:** Regression results for the association between long-term exposure to ambient air pollution and HOMA-IR.

Characteristics	HOMA-IR							
	Crude		Model 1^a^		Model 2^b^		Model 3^c^	
	*β* (95% CI)	*p* value	*β* (95% CI)	*p* value	*β* (95% CI)	*p* value	*β* (95% CI)	*p* value
**Total**								
PM_10_ (μg/m^3^)	0.18 (0.13, 0.24)	< 0.001	0.16 (0.11, 0.21)	< 0.001	0.15 (0.10, 0.20)	< 0.001	0.15 (0.10, 0.20)	< 0.001
NO_2_ (ppb)	0.02 (–0.03, 0.06)	0.450	0.02 (–0.02, 0.06)	0.269	0.01 (–0.03, 0.05)	0.565	0.01 (–0.03, 0.05)	0.540
SO_2_ (ppb)	0.01 (–0.05, 0.06)	0.775	0.01 (–0.04, 0.06)	0.767	0.01 (–0.04, 0.06)	0.624	0.01 (–0.04, 0.06)	0.596
CO (ppm)	0.09 (0.03, 0.15)	0.002	0.06 (0.01, 0.11)	0.015	0.05 (0.00, 0.10)	0.044	0.05 (0.00, 0.10)	0.040
**Men**								
PM_10_ (μg/m^3^)	0.17 (0.10, 0.24)	< 0.001	0.15 (0.09, 0.22)	< 0.001	0.15 (0.08, 0.21)	< 0.001	0.14 (0.08, 0.21)	< 0.001
NO_2_ (ppb)	0.01 (–0.04, 0.07)	0.606	0.02 (–0.03, 0.08)	0.349	0.01 (–0.04, 0.06)	0.588	0.02 (–0.03, 0.07)	0.507
SO_2_ (ppb)	–0.01 (–0.09, 0.06)	0.677	–0.03 (–0.09, 0.04)	0.417	–0.01 (–0.08, 0.05)	0.664	–0.02 (–0.08, 0.05)	0.590
CO (ppm)	0.08 (0.00, 0.15)	0.039	0.06 (–0.00, 0.13)	0.062	0.05 (–0.01, 0.12)	0.099	0.06 (–0.01, 0.12)	0.090
**Women**								
PM_10_ (μg/m^3^)	0.22 (0.14, 0.29)	< 0.001	0.16 (0.09, 0.23)	< 0.001	0.14 (0.07, 0.21)	< 0.001	0.14 (0.07, 0.21)	< 0.001
NO_2_ (ppb)	0.02 (–0.06, 0.09)	0.675	0.02 (–0.04, 0.09)	0.526	0.01 (–0.06, 0.07)	0.847	0.01 (–0.06, 0.07)	0.857
SO_2_ (ppb)	0.07 (–0.01, 0.15)	0.085	0.07 (–0.00, 0.14)	0.052	0.06 (–0.01, 0.13)	0.120	0.05 (–0.02, 0.12)	0.140
CO (ppm)	0.13 (0.04, 0.21)	0.003	0.07 (–0.00, 0.15)	0.059	0.05 (–0.02, 0.13)	0.168	0.05 (–0.02, 0.12)	0.190

*HOMA-IR* homeostasis model assessment of insulin resistance index, *PM*_*10*_ particulate matter with aerodynamic diameter ≤ 10 μm, *NO*_*2*_ nitrogen dioxide, *SO*_*2*_ sulfur dioxide, *CO* carbon monoxide, *CI* confidence interval.

The beta coefficients and 95% CIs of air pollutants were scaled to the interquartile range (IQR) for each air pollutant: 11 μg/m^3^ for PM_10_, 14 ppb for NO_2_, 2 ppb for SO_2_, and 0.2 ppm for CO.

^a^Model 1 was adjusted for age, BMI, smoking status (never, former, or current smoking), alcohol consumption (never, former, or current consumption), regular physical activity (yes or no), hypertension, and dyslipidemia.

^b^Model 2 included the variables in Model 1 plus VAT and SAT areas as covariates.

^c^Model 3 included the variables in Model 1 plus VAT area as a covariate.

**Table 4:** Regression results for the association between exposure to ambient air pollution and the presence of insulin resistance.

Characteristic	Presence of insulin resistance							
	Crude		Model 1^a^		Model 2^b^		Model 3^c^	
	OR (95% CI)	*p* value	OR (95% CI)	*p* value	OR (95% CI)	*p* value	OR (95% CI)	*p* value
**Total**								
PM_10_ (μg/m^3^)	1.29 (1.17, 1.43)	< 0.001	1.29 (1.16, 1.44)	< 0.001	1.27 (1.14, 1.42)	< 0.001	1.27 (1.14, 1.42)	< 0.001
NO_2_ (ppb)	1.02 (0.94, 1.11)	0.595	1.05 (0.95, 1.15)	0.346	1.03 (0.93, 1.13)	0.592	1.03 (0.94, 1.13)	0.547
SO_2_ (ppb)	0.99 (0.90, 1.10)	0.899	1.00 (0.89, 1.11)	0.953	1.00 (0.90, 1.12)	0.952	1.01 (0.9., 1.12)	0.909
CO (ppm)	1.15 (1.04, 1.28)	0.007	1.13 (1.01, 1.27)	0.031	1.11 (0.99, 1.24)	0.071	1.11 (0.99, 1.25)	0.062
**Men**								
PM_10_ (μg/m^3^)	1.19 (1.06, 1.35)	0.005	1.20 (1.05, 1.37)	0.008	1.18 (1.03, 1.35)	0.016	1.18 (1.03, 1.35)	0.017
NO_2_ (ppb)	1.01 (0.92, 1.12)	0.815	1.04 (0.94, 1.16)	0.439	1.02 (0.92, 1.14)	0.658	1.03 (0.93, 1.15)	0.558
SO_2_ (ppb)	0.93 (0.82, 1.06)	0.272	0.92 (0.80, 1.05)	0.206	0.93 (0.81, 1.07)	0.309	0.93 (0.81, 1.06)	0.285
CO (ppm)	1.1. (0.97, 1.25)	0.121	1.11 (0.97, 1.27)	0.121	1.10 (0.96, 1.26)	0.170	1.10 (0.96, 1.26)	0.151
**Women**								
PM_10_ (μg/m^3^)	1.56 (1.30, 1.86)	< 0.001	1.49 (1.22, 1.81)	< 0.001	1.44 (1.18, 1.76)	< 0.001	1.44 (1.18, 1.76)	< 0.001
NO_2_ (ppb)	1.04 (0.88, 1.23)	0.644	1.06 (0.88, 1.26)	0.548	1.02 (0.85, 1.23)	0.795	1.02 (0.85, 1.23)	0.800
SO_2_ (ppb)	1.18 (0.98, 1.41)	0.086	1.21 (0.99, 1.47)	0.063	1.17 (0.96, 1.43)	0.124	1.17 (0.96, 1.42)	0.130
CO (ppm)	1.29 (1.06, 1.55)	0.009	1.18 (0.96, 1.45)	0.125	1.13 (0.91, 1.39)	0.269	1.12 (0.91, 1.38)	0.283

*HOMA-IR* homeostasis model assessment of insulin resistance index, *PM*_*10*_ particulate matter with aerodynamic diameter ≤ 10 μm, *NO*_*2*_ nitrogen dioxide, *SO*_*2*_ sulfur dioxide, *CO* carbon monoxide, *OR* odds ratio, *CI* confidence interval.

The ORs and 95% CIs of air pollutants were scaled to the interquartile range (IQR) for each air pollutant: 11 μg/m^3^ for PM_10_, 14 ppb for NO_2_, 2 ppb for SO_2_, and 0.2 ppm for CO.

^a^Model 1 was adjusted for age, BMI, smoking status (never, former, or current smoking), alcohol consumption (never, former, or current consumption), regular physical activity (yes or no), hypertension and dyslipidemia.

^b^Model 2 included the variables in Model 1 plus VAT and SAT areas as covariates.

^c^Model 3 included the variables in Model 1 plus VAT area as a covariate.

**Figure 1:**
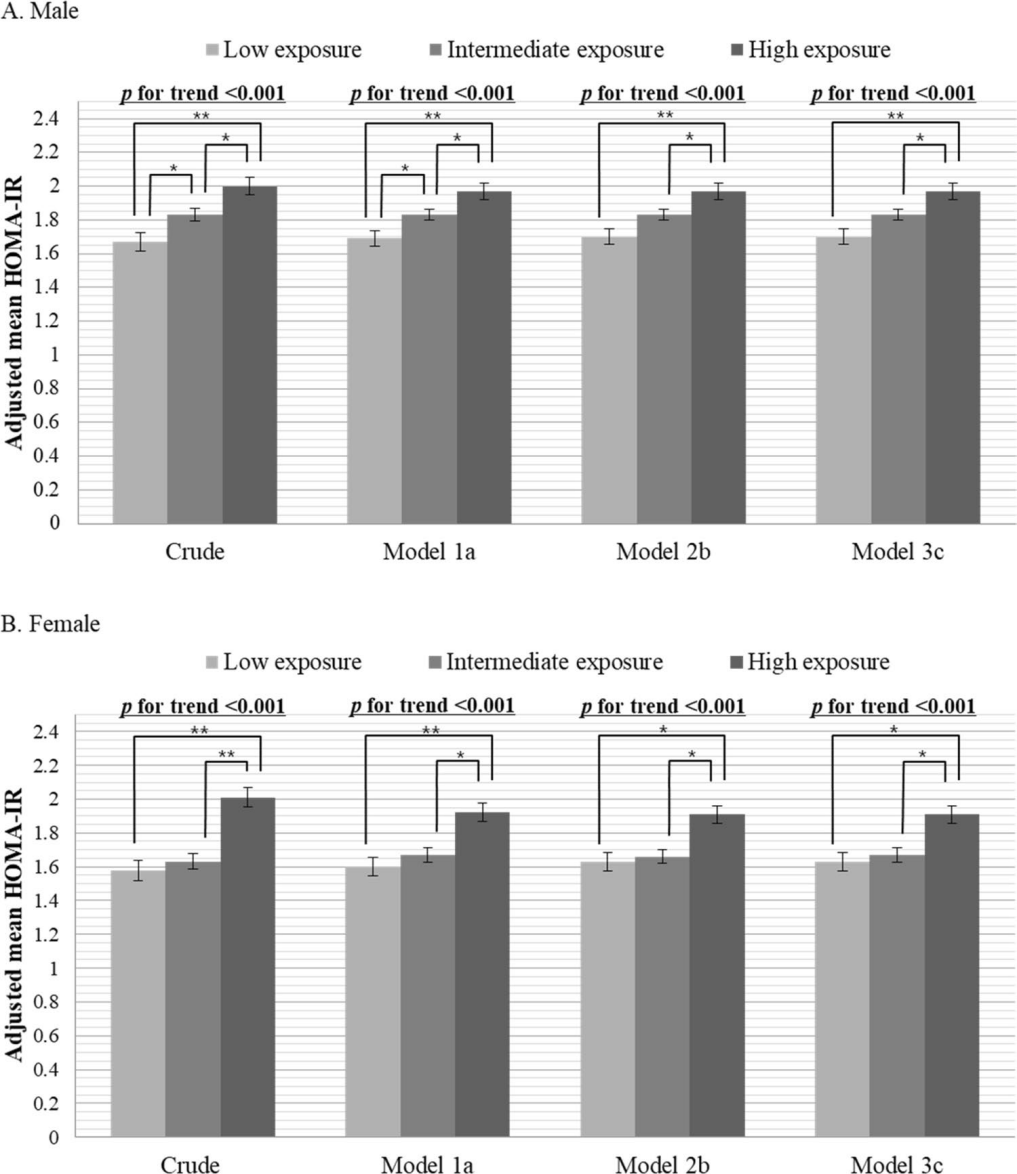
Adjusted mean HOMA-IR according to long-term PM_10_ exposure. Adjusted mean HOMA-IR was calculated using linear regression analysis. Exposure to PM_10_ was classified using quartiles of PM_10_ concentration as low exposure (quartile 1: mean = 40.22, SD = 3.29), intermediate exposure (quartiles 2 and 3: mean = 49.19, SD = 2.95), and high exposure (quartile 4: mean = 60.63, SD = 4.91). *p < 0.05, **p < 0.001. ^a^Model 1 was adjusted for age, BMI, smoking status (never, former, or current smoking), alcohol consumption (never, former, or current consumption), regular physical activity (yes or no), hypertension, and dyslipidemia. ^b^Model 2 included the variables in Model 1 plus VAT and SAT areas as covariates. ^c^Model 3 included the variables in Model 1 plus VAT area as a covariate. *HOMA-IR* homeostasis model assessment of insulin resistance index, *PM*_*10*_ particulate matter with aerodynamic diameter ≤ 10 μm^3^, *BMI* body mass index, *VAT* visceral adipose tissue, *SAT* subcutaneous adipose tissue.

The increase in the stratified exposure to PM_10_ was independently associated with higher HOMA-IR in a dose-dependent manner in both men (1.70, 1.83 and 1.97 in Model 3) and women (1.63, 1.67 and 1.91 in Model 3) (both *p* for trend < 0.001) (Fig. 1). The mean concentrations of PM_10_ were 40.22 (SD = 3.29), 49.19 (SD = 2.95), and 60.63 (SD = 4.91) in the low, intermediate, and high exposure groups, respectively.

### Associations between long-term exposure to AP and the presence of IR

The associations between long-term exposure to AP and the presence of IR are shown in Table 4. In all crude and adjusted models, an increase in the IQR for PM_10_ exposure was associated with elevated odds for the presence of IR in both men (OR = 1.18; 95% CI = 1.03, 1.35 in Model 3) and women (OR = 1.44; 95% CI = 1.18, 1.76 in Model 3). Exposure to other pollutants was not significantly associated with the presence of IR in the adjusted models that included VAT area as a covariate (Models 2 and 3).

## Discussion

This study investigated the associations between exposure to AP and IR in the general population of Korean adults and whether these associations were maintained after controlling for the effects of abdominal fat, particularly visceral fat. We also examined whether such relationships are related to the dose of PM_10_ exposure. We observed that there was no significant association between exposure to NO_2_, SO_2_ or CO and HOMA-IR or the presence of IR but that long-term higher exposure to PM_10_ was associated with an increasing trend for both HOMA-IR and the presence of IR in a dose-dependent manner regardless of BMI or abdominal fat level, as measured by VAT and SAT areas.

Our findings are consistent with those of a recent systematic review and meta-analysis that reported positive correlations between PM_10_ exposure and IR, as assessed using HOMA-IR^19^. The first study reported in 2013 involved 10-year-old children in two German birth cohort studies and showed positive correlations between PM_10_ exposure and HOMA-IR^20^. Another study from Germany in 2016 also reported a positive association between PM_10_ exposure and HOMA-IR in prediabetic patients^14^. A study conducted in China in 2018 found similar associations in younger adults aged < 50 years^18^. However, other studies of the associations between PM_10_ and IR have reported inconsistent results, possibly because of the diverse population characteristics, such as age, sex or diabetic state^14,18,23^.

A lack of significant associations has been reported in studies of patients with DM^14^, in elderly populations^23^, and when assessing short-term exposure to PM_10_^23^. Additionally, most of these studies were conducted in Germany or the United States, where the mean concentration of PM_10_ of approximately 20 μm/m^3^ is lower than the level of 49.67 μm/m^3^ (SD = 7.85) in our study of the Korean population. We found a positive correlation between PM_10_ exposure and IR, and this association was dose dependent. The dose-dependent association and higher AP exposure found in our study lead us to speculate that the lack of significant associations in earlier studies may have been caused by the low AP exposure in those studies. Moreover, insulin sensitivity varies between ethnic groups, and Asians are more susceptible to IR than are people of European descent, which suggests that differences in results may reflect differences in the ethnicity of the populations studied. Our study results support the concept of a positive relationship between PM_10_ exposure and IR in the general Korean population.

On the other hand, the finding of no significant association between NO_2_ and IR in our study contrasts with some previous reports of a positive correlation between these variables^14,17–19,23,35^. However, the difference may also be attributed to the different settings of each study, such as the assessment of short-term exposure to NO_2_ or specific populations, such as prediabetes patients, obese youth, or younger adults aged < 50 years^23^. A study of the general population in China showed no significant association between AP exposure and HOMA-IR^18^, as seen in our study. It is possible that NO_2_ exposure is not related to IR in the general Asian population.

Abdominal fat, especially VAT, is a major contributor to IR development and was significantly associated with IR in our study. Compared with BMI or SAT area, VAT area was more strongly associated with IR, and the correlation was stronger in women than in men. Although waist circumference (WC) and waist-to-hip ratio (WHR) are more important risk factors for type 2 DM than overall adiposity, these measures cannot distinguish VAT from SAT. VAT is higher for a given BMI or WC in South Asian men and women than in Europeans^36,37^. Therefore, considering only BMI or WC does not reflect the possible confounding effect of VAT on the association between AP exposure and IR.

Despite strong evidence of the effects of VAT on IR and the limited correlation between BMI and VAT, previous studies have not considered the effects of VAT on the association between AP exposure and IR. Most studies have considered only BMI or percent body fat as a covariate^13,16,18,19^, and some have adjusted for WHR^14,24^. Therefore, it was not clear whether AP exposure leads to IR through the intermediate of increased abdominal fatness or whether AP exposure leads to IR independent of abdominal fatness. In our models that included VAT as a covariate, we found that higher long-term PM_10_ exposure was associated with both HOMA-IR and the presence of IR regardless of abdominal fatness, including that reflected by VAT area. These associations suggest that the mechanism underlying the effects of long-term PM_10_ exposure on IR development is independent of fat metabolism. Although the mechanism underlying the relationship between AP and IR remains unclear, previous studies have suggested several pathways, including inflammation and oxidative stress^21,22^. Inhaled air pollutants may induce pulmonary and systemic inflammation and endoplasmic reticulum stress, which may then alter insulin signaling and glucose metabolism and eventually cause IR^21,22^.

Our study has a number of strengths. This is the first study to measure abdominal fatness (VAT and SAT areas) by CT to examine the associations between long-term AP exposure and IR. In addition, it was conducted in a large sample of the general population. We recorded the concentrations of PM_10_ at approximately 300 atmospheric monitoring sites nationwide to obtain more precise estimates of the PM_10_ exposure level of each participant. However, our study also has some limitations. First, we did not identify people with DM who were taking insulin sensitizers such as metformin or thiazolidinedione, which may affect IR. Second, the AP levels for each participant were estimated according to their registered address, which did not account for the possible diversity of exposure, such as that related to occupation, indoor and outdoor activity, and the areas of employment area and residence. Third, we used a cross-sectional study design. However, because PM_10_ exposure is not influenced by IR, it is reasonable to expect that long-term exposure to PM_10_ affects the development of IR and not vice versa.

## Conclusions

We examined the associations between long-term exposure to AP and the presence of IR. We found that long-term higher exposure to PM_10_ was independently associated with both the mean HOMA-IR and the presence of IR in a dose-dependent manner, and the association was independent of abdominal fatness in both men and women.

## Supplementary Information

Supplementary Information.

## Data Availability

The data that support the findings of this study are available from SNUH, but restrictions apply to the availability of these data, which were used under license for the current study and thus are not publicly available. Data are, however, available from the author, Jin-Ho Park upon reasonable request and with permission of SNUH.

## Abbreviations

AP: Air pollution
ASCVD: Atherosclerotic cardiovascular disease
BMI: Body mass index
BP: Blood pressure
CI: Confidence interval
CO: Carbon monoxide
CT: Computed tomography
DM: Diabetes mellitus
HOMA-IR: Homeostasis model assessment of insulin resistance
IQR: Interquartile range
IR: Insulin resistance
NO_2_: Nitrogen dioxide
OR: Odds ratio
PM_10_: Particulate matter with an aerodynamic diameter ≤ 10 mm
PM_2.5_: Particulate matter with an aerodynamic diameter ≤ 2.5 mm
ppb: Parts per billion
SAT: Subcutaneous adipose tissue
SD: Standard deviation
SNUH: Seoul National University Hospital
SO_2_: Sulfur dioxide
TAT: Total adipose tissue
VAT: Visceral adipose tissue
WC: Waist circumference
WHR: Waist-to-hip ratio
