# Supplementary material for: Association between long-term air pollution exposure and insulin resistance independent of abdominal adiposity in Korean adults
Source: Sci Rep. 2022 Nov 9;12:19147. doi: 10.1038/s41598-022-23324-4 (PMC9646867; doi:10.1038/s41598-022-23324-4)
Supplement: Supplementary file 1 — Supplementary Information. [file 41598_2022_23324_MOESM1_ESM.pptx]

## Slide 1
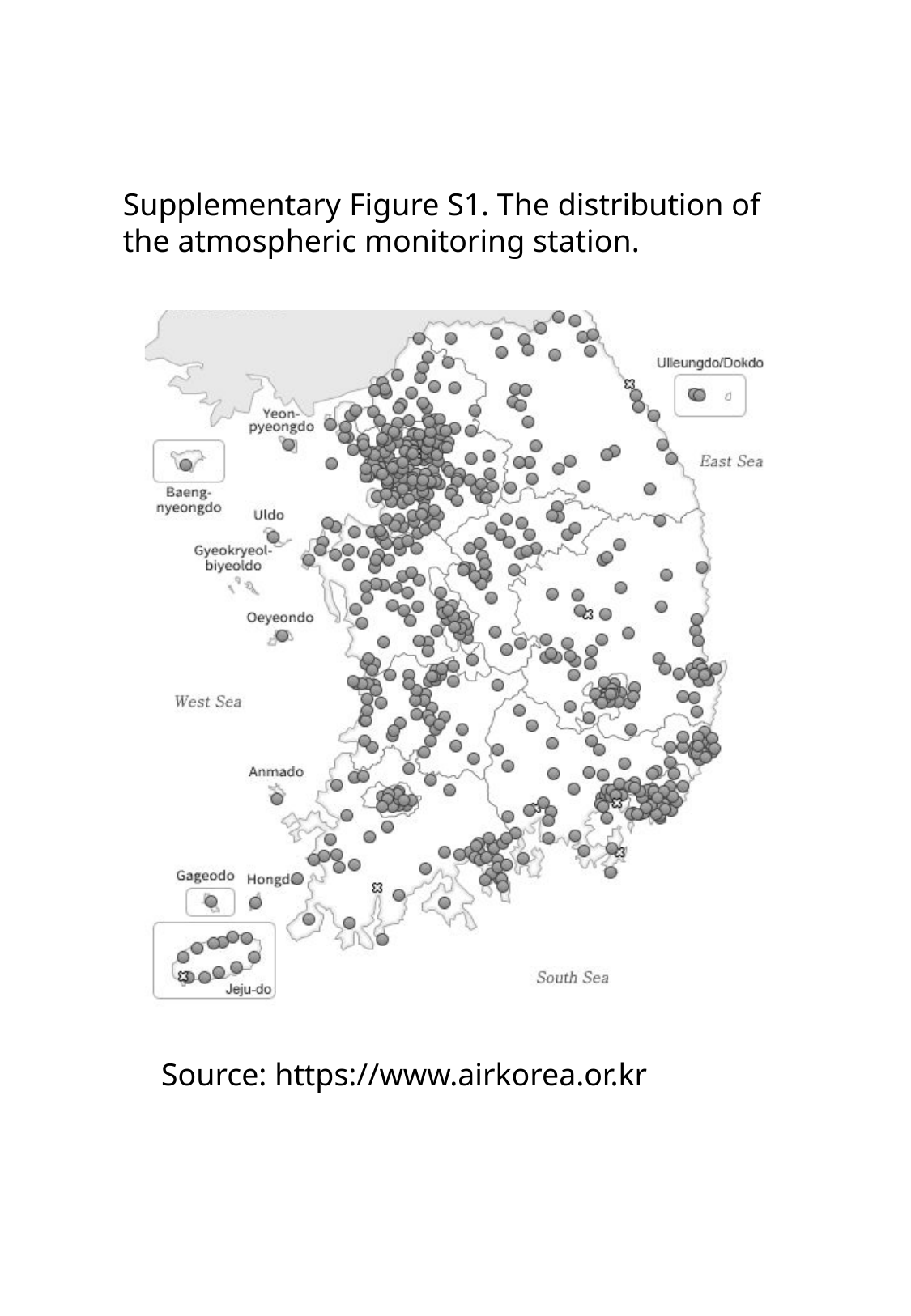

Supplementary Figure S1. The distribution of the atmospheric monitoring station.
Source: https://www.airkorea.or.kr
